# Common polymorphisms in human lysyl oxidase genes are not associated with the adolescent idiopathic scoliosis phenotype

**DOI:** 10.1186/1471-2350-12-92

**Published:** 2011-07-08

**Authors:** Tracy L McGregor, Christina A Gurnett, Matthew B Dobbs, Carol A Wise, Jose A Morcuende, Thomas M Morgan, Ramkumar Menon, Louis J Muglia

**Affiliations:** 1 Department of Pediatrics, Vanderbilt University School of Medicine, Nashville, TN 37232 USA; 2 Center for Human Genetics Research, Vanderbilt University School of Medicine, Nashville, TN 37232 USA; 3 Department of Neurology, Washington University School of Medicine, St. Louis, MO 63110 USA; 4 Department of Pediatrics, Washington University School of Medicine, St. Louis, MO 63110 USA; 5 Department of Orthopedic Surgery, Washington University School of Medicine, St. Louis, MO 63110 USA; 6 St. Louis Shriners Hospital for Children, St. Louis, MO 63131 USA; 7 Sarah M. and Charles E. Seay Center for Musculoskeletal Research, Texas Scottish Rite Hospital for Children, Dallas, TX 75219 USA; 8 Department of Orthopaedic Surgery, University of Texas Southwestern Medical Center, Dallas, TX 75390 USA; 9 McDermott Center for Human Growth and Development, University of Texas Southwestern Medical Center, Dallas, TX 75390 USA; 10 Department of Orthopedic Surgery and Rehabilitation, University of Iowa, Iowa City, IA 52242 USA; 11 The Perinatal Research Center, Nashville, TN, 37203 USA; 12 Department of Epidemiology, Rollins School of Public Health, Emory University, Atlanta, GA, 30322 USA; 13 Vanderbilt Kennedy Center for Human Development, Vanderbilt University, Nashville, TN 37232 USA; 14 Department of Molecular Physiology and Biophysics, Vanderbilt University School of Medicine, Vanderbilt University, Nashville, TN 37232 USA

## Abstract

**Background:**

Although adolescent idiopathic scoliosis affects approximately 3% of adolescents, the genetic contributions have proven difficult to identify. Work in model organisms, including zebrafish, chickens, and mice, has implicated the lysyl oxidase family of enzymes in the development of scoliosis. We hypothesized that common polymorphisms in the five human lysyl oxidase genes (*LOX, LOXL1, LOXL2, LOXL3*, and *LOXL4*) may be associated with the phenotype of adolescent idiopathic scoliosis.

**Methods:**

This was a case-control genetic association study. A total of 112 coding and tag SNPs in *LOX, LOXL1, LOXL2, LOXL3*, and *LOXL4 *were genotyped in a discovery cohort of 138 cases and 411 controls. Genotypes were tested for association with adolescent idiopathic scoliosis by logistic regression with a two degree of freedom genotypic model and gender as a covariate. Fourteen SNPs with p < 0.1 in the discovery phase were genotyped in an independent replication cohort of 400 cases and 506 controls.

**Results:**

No evidence for significant association was found between coding or tag SNPs in *LOX*, *LOXL1*, *LOXL2*, *LOXL3*, and *LOXL4 *and the phenotype of adolescent idiopathic scoliosis.

**Conclusions:**

Despite suggestive evidence in model organisms, common variants and known coding SNPs in the five human lysyl oxidase genes do not confer increased genotypic risk for adolescent idiopathic scoliosis. The above methodology does not address rare variants or individually private mutations in these genes, and future research may focus on this area.

## Background

Adolescent idiopathic scoliosis (AIS) affects 2-3% of the pediatric population [1] and often requires bracing or surgical treatment. AIS is currently recognized as a multifactorial disease with multiple influences, both environmental and genetic [2,3]. Multiple attempts have been made to identify the genetic etiologies of AIS with only limited success, despite evidence for genetic contributions.

Multiple studies have made a strong case for heritability of the incidence of AIS. A meta-analysis of 68 sets of twins found concordance in 73% of monozygous twins and 36% of dizygous twins [4]. A more recent study relying on self report survey methodology confirmed the increased concordance in monozygotic twin pairs (6/44) relative to dizygotic pairs (0/91) [5]. The search for underlying genes has only uncovered a few viable candidates, namely *SNTG1 *[6] and *CHD7 *[7]. Additionally, a recent genome-wide agnostic approach put forward a promising finding in *CHL1*, but the authors were unable to replicate the association in all independent populations [8].

The human lysyl oxidases are a family of copper-dependent enzymes involved in the modeling of connective tissue. These enzymes oxidize the side chain of peptidyl lysine converting specific lysine residues to residues of α-aminoadipic-d-semialdehyde, allowing crosslinking of collagen and elastin. The lysyl oxidase enzymes employ a copper ion (Cu^2+^) as one of the essential cofactors [9]. This family of enzymes has been proposed to play a role in a range of human diseases including exfoliation glaucoma [10], myocardial fibrosis [11], intracranial aneurysms [12] and cancer metastases [13].

Research in model organisms has linked lysyl oxidase activity with scoliotic phenotypes. Zebrafish researchers have shown that disrupting lysyl oxidase activity in embryonic fish results in notochord distortion leading to defects of the axial skeleton [14]. Work in a line of chickens susceptible to scoliosis implicated the lysyl oxidases as a causative feature [15,16], but this has not yet been definitively shown. Recent work has investigated the murine lysyl oxidases with bone development [17-19], but a scoliotic phenotype has not been specifically studied in a murine model.

Specific investigations of human lysyl oxidases and scoliosis have been limited to hypotheses generated by findings of increased copper content in hair and plasma samples from scoliotic patients [20-22]. To our knowledge, the relationship between adolescent idiopathic scoliosis and variants in the lysyl oxidase family of genes has not been investigated. This study was designed to test for association between common polymorphisms in the five human lysyl oxidase genes with the phenotype of adolescent idiopathic scoliosis.

## Methods

### Sample Size

Power calculations were performed prior to initiation of the study with the Genetic Power Calculator [23]. As expected, the genotypic relative risk detectable with our estimated discovery cohort sample size of 180 cases and 360 controls varied with allele frequency over the range of 0.05 to 0.35. Our anticipated detectable difference in the discovery cohort ranged from an odds ratio of 1.7 to 2.0. Parameters included the 2 degree of freedom genotypic test with alpha = 0.1 and 80% power; assumptions included genotyping the causative SNP, a dominant model without additive or multiplicative effects, and a population prevalence of 3%.

### Populations

#### Discovery Cohort

Patients with AIS collected at Washington University School of Medicine, St. Louis Children's Hospital, and Shriner's Hospital for Children in St. Louis, MO were genotyped as cases in the discovery cohort. Inclusion criteria for this cohort were spinal curvature >10 degrees on radiograph and Caucasian ancestry. Exclusion criteria were known or suspected associated diagnoses such as Marfan syndrome or neuromuscular disease. Patients provided written informed consent as part of an IRB protocol approved by Washington University School of Medicine and Shriner's Hospital for Children. DNA was isolated from either lymphocytes or saliva via standard procedures. An aliquot of each sample was then whole genome amplified with the QIAGEN REPLI-g kit per the manufacturer's directions (Valencia, CA). Amplification was confirmed by agarose gel electrophoresis (data not shown).

The population control patients for the discovery cohort were recruited at an ambulatory outpatient clinical laboratory in Kansas City, MO as previously described [24]. Patients provided written informed consent as part of an IRB protocol approved by Saint Luke's Hospital of Kansas City. Scoliosis status was not assessed clinically or radiographically. Ancestry and gender information was obtained by self-report. Only participants identified as Caucasian were included in this study, and controls were frequency matched for gender. DNA was previously extracted from lymphocytes by standard procedures and whole genome amplified via QIAGEN REPLI-g per the manufacturer's directions (Valencia, CA).

#### Replication Cohort

A portion of the replication cohort cases (n = 130) were recruited at the University of Iowa. Inclusion criteria were Cobb angle of at least 10 degrees with pedicle rotation by radiograph and Caucasian ancestry. Exclusion criteria were evidence of neuromuscular or congenital scoliosis, or other recognizable syndromes involving scoliosis. The remainder of the cases in the replication cohort (n = 270) were collected at Texas Scottish Rite Hospital in Dallas, TX. Inclusion criteria were scoliosis of at least 15 degrees by radiograph and Caucasian ancestry. Exclusion criteria were neuromuscular, congenital, or syndromic scoliosis, or family history of the same. In both populations, genomic DNA was isolated from whole blood or saliva by standard procedures and resuspended in water. All patients provided written informed consent as part of IRB protocols approved by the University of Iowa and University of Texas Southwestern Medical Center and Texas Scottish Rite Hospital.

Population control patients were obtained from patients recruited in a clinical laboratory in Kansas City, MO, as described above, without overlap of individuals between cohorts. Women recruited by the Perinatal Research Center in Nashville, TN also served as controls. These participants provided written informed consent as part of a protocol approved by TriStar Nashville IRB, Nashville, TN and Western IRB, Seattle, WA. Scoliosis status was not assessed clinically or radiographically. Inclusion criterion was Caucasian ancestry, obtained by self report. The gender ratio of controls was again frequency matched to that of the cases in the replication cohort.

A summary of the populations is given in Table 1. The ages of the controls are more advanced than those of cases, indicating that they were beyond the age of developing the AIS phenotype. We maintained the assumption of 3% prevalence of AIS in the control populations, as we would not expect AIS to increase the chances of inclusion in the control populations.

**Table 1:** Description of populations genotyped in discovery and replication phases

Collection site	N	Cohort	Minimum curvature	Percent female	Average Age (y)
**St. Louis, MO**	**138**	Discovery	10 degrees	85.5	16.9 ± 6.2
**Kansas City, MO**	**411**	Discovery	Population Control	85.0	61.5 ± 12.7
**Dallas, TX**	**270**	Replication	15 degrees	87.0	14.6 ± 2.4
**Iowa City, IA**	**130**	Replication	10 degrees	84.5	22.9 ± 15.7
**Kansas City, MO**	**179**	Replication	Population Control	68.7	61.2 ± 12.6
**Nashville, TN**	**327**	Replication	Population Control	100.0	28.5 ± 5.8

The geographic location where each cohort was enrolled as well as details about the populations are presented.

### SNP Selection

All SNPs from the lysyl oxidase genes denoted as coding in dbSNP build 129 [25] were candidates for genotyping regardless of the minor allele frequency. HapMap data [26] from the CEU population was analyzed into the Haploview (version 2) program [27] for 100 kb upstream and downstream of the five genes studied, *LOX, LOXL1, LOXL2, LOXL3 *and *LOXL4*. The Tagger algorithm [28] selected tag SNPs with the following parameters: pairwise tagging only; r^2 ^threshold 0.8; MAF cutoff 0.1; Design score 1. The lists of coding SNPs were marked as included SNPs in the Tagger algorithm and no SNPs were excluded *a priori*. This resulting list was submitted to the Vanderbilt DNA Resources Core for genotyping on the Sequenom MassARRAY system (San Diego, CA). After assay design with proprietary Sequenom software, those tag SNPs which failed probe design or did not pool with other markers were added to the excluded SNPs list, and iterations performed until all regions were tagged. SNPs that associated with AIS after controlling for gender with p < 0.1 prior to multiple test correction were carried forward to replication genotyping.

### Genotyping

The Vanderbilt DNA Resources Core performed the genotyping with the use of the Sequenom MassARRAY system (San Diego, CA). This technology is based on a single-base primer extension reaction coupled with mass spectrometry. Quality-control procedures included examination of marker and sample genotyping efficiency, allele frequency calculations, accuracy of known HapMap samples, and testing of Hardy-Weinberg equilibrium.

### Analysis

Genetic analyses including Hardy Weinberg equilibrium testing, allele frequency, logistic regression, and allele association were performed with gPLINK v2.050 [29,30]. SNPs with total genotyping efficiency of <85% were excluded from further analysis. Individual samples with genotyping efficiency of <70% were also excluded. Allele frequencies were calculated in both cases and controls, and were analyzed for differences between cohorts. Genotype results were tested for deviation from Hardy-Weinberg equilibrium in control samples.

Genotypes in both cohorts were analyzed by multivariate logistic regression with gender as a covariate and case/control status as the outcome. The analyses did not assume a prespecified genetic model and was conducted with a genotypic model requiring two degrees of freedom. In the discovery cohort, the SNPs with p-values <0.1 for the overall model prior to multiple test correction were carried forward for genotyping in the replication cohort. In the replication cohort, stratified analyses were conducted to ensure that the origin of the samples or gender of the subject did not act as a confounder or effect modifier. As a secondary analysis, the results of an allele association test requiring only 1 degree of freedom was performed.

## Results

For initial SNP selection, the Tagger algorithm initially identified a total of 132 candidate coding and tag SNPs. After reiterations to accommodate the Sequenom Genotyping platform, final selection included 112 SNPs. These were genotyped in 138 case and 411 control samples (Additional File 1). Of these, 9 SNPs and 15 samples (8 cases and 7 controls) failed genotyping and were excluded from further analyses. A total of 16 SNPs in *LOXL1*, *LOXL2*, and *LOXL4 *(Table 2) showed a difference by genotype with an uncorrected adjusted p < 0.1. These SNPs were carried forward for genotyping in the replication population.

**Table 2:** SNPs genotyped in discovery cases and controls with p <0.1

Gene	SNP	Minor allele and strand	Logistic regression adjusted p value	Genotypic association OR (95% CI) Heterozygous (Het) Homozygous minor (HM)	Genotype Frequencies minor/het/major Cases (Ca) Pop. Controls (PC)
***LOXL1***	rs12442211	G/-	0.066	Het: 0.60 (0.37 - 0.95)	Ca: 27/54/47
				HM: 0.60 (0.34 - 1.04)	PC: 100/198/101
***LOXL1***	rs2304719	T/-	0.063	Het: 0.61 (0.39 - 0.96)	Ca: 17/39/69
				HM: 1.12 (0.59 - 2.14)	PC: 40/171/189
***LOXL1***	rs4461027	C/-	0.058	Het: 0.59 (0.38 - 0.91)	Ca: 23/46/62
				HM: 0.72 (0.41 - 1.26)	PC: 71/186/142
***LOXL1***	rs4886782	A/-	0.031	Het: 0.56 (0.36 - 0.86)	Ca: 18/43/69
				HM: 0.86 (0.46 - 1.58)	PC: 51/186/161
***LOXL2***	rs1002791	C/+	0.039	Het: 0.58 (0.38 - 0.90)	Ca: 6/39/82
				HM: 0.58 (0.23 - 1.47)	PC: 26/166/208
***LOXL2***	rs17760913	T/-	0.055	Het: 0.66 (0.42 - 1.04)	Ca: 12/34/86
				HM: 1.62 (0.76 - 3.46)	PC: 21/142/227
***LOXL2***	rs17760943	A/-	0.072	Het: 0.77 (0.49 - 1.19)	Ca: 16/39/76
				HM: 1.75 (0.89 - 3.44)	PC: 28/150/222
***LOXL2***	rs2294125	G/+	0.10	Het: 0.67 (0.41 - 1.08)	Ca: 36/52/41
				HM: 1.09 (0.64 - 1.85)	PC: 88/201/107
***LOXL2***	rs3808522	G/-	0.0046	Het: 0.48 (0.28 - 0.82)	Ca: 37/30/46
				HM: 1.15 (0.68 - 1.94)	PC: 80/150/111
***LOXL2***	rs3808536	C/+	0.0059	Het: 0.69 (0.42 - 1.11)	Ca: 40/47/40
				HM: 1.57 (0.92 - 2.68)	PC: 73/200/119
***LOXL2***	rs6985160	T/-	0.035	Het: 0.59 (0.38 - 0.90)	Ca: 14/47/68
				HM: 0.59 (0.30 - 1.13)	PC: 55/185/155
***LOXL2***	rs6999447	T/-	0.027	Het: 0.86 (0.55 - 1.35)	Ca: 29/50/53
				HM: 1.85 (1.06 - 3.24)	PC: 48/182/170
***LOXL4***	rs11189510	A/+	0.00045	Het: 2.30 (1.45 - 3.66)	Ca: 5/39/87
				HM: 3.84 (1.09-13.58)	PC: 6/68/325
***LOXL4***	rs11189513	G/-	0.036	Het: 1.23 (0.81 - 1.87)	Ca: 9/60/60
				HM: 0.45 (0.21 - 0.97)	PC: 63/151/183
***LOXL4***	rs11599085	C/+	0.067	Het: 1.69 (1.09 - 2.64)	Ca: 17/76/39
				HM: 1.39 (0.72 - 2.66)	PC: 53/185/164
***LOXL4***	rs751160	G/-	0.093	Het: 0.69 (0.44 - 1.07)	Ca: 22/48/59
				HM: 1.26 (0.69 - 2.30)	PC: 47/190/157

The SNPs genotyped in the discovery phase (138 cases and 411 controls) with overall p <0.1 are represented. The minor allele and strand are represented to allow for comparisons between studies. The p-value displayed indicates the significance of the overall model and is adjusted for gender. The odds ratios use the homozygous major allele genotype as the reference genotype and genotype counts are presented for each group.

**Figure 1:**
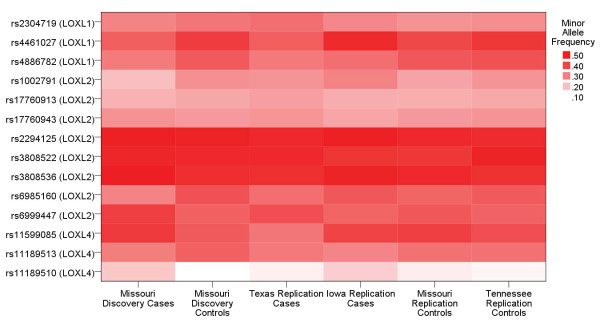
**Minor allele frequency comparison between populations**. The minor allele frequencies of the SNPs successfully genotyped in the replication phase are represented. The minor allele frequency did not differ significantly between the six populations as assessed by chi squared analysis.

Of the 16 SNPs carried forward to the replication samples, rs751160 (*LOXL4*) and rs12442211 (*LOXL1*) were not successfully genotyped. In the cohort of 400 cases and 506 controls, 17 samples (7 cases and 10 controls) failed genotyping. A total of 14 SNPs were analyzed within the replication cohort using the same two degree of freedom genotypic model with multivariate logistic regression, adjusting for gender. The minor allele frequencies were consistent between populations (Figure 1). No SNPs showed a difference by genotype between cases and controls with p < 0.004 (Table 3). Because the cases in the replication cohort were obtained from two different recruiting centers, the analysis was stratified by recruiting center to assess for confounding. No SNP attained significance in a single cohort that was masked by combining the analysis. In addition, the analysis was stratified by gender to determine if gender was acting as an effect modifier. No significant associations or evidence for effect modification were detected.

**Table 3:** Significance and odds ratios of SNPs genotyped in the replication phase

Gene	SNP	Minor allele and strand	Logistic regression adjusted p value	Genotypic association OR (95% CI) Heterozygous (Het) Homozygous minor (HM)	Genotype Frequencies minor/het/major Cases (Ca) Pop. Controls (PC)
***LOXL1***	rs2304719	T/-	0.2	Het: 1.19 (0.90 - 1.57)	Ca: 36/172/184
				HM: 1.51 (0.91 - 2.52)	PC: 33/202/255
***LOXL1***	rs4461027	C/-	0.19	Het: 1.01 (0.76 - 1.36)	Ca: 55/190/148
				HM: 0.72 (0.48 - 1.07)	PC: 91/224/177
***LOXL1***	rs4886782	A/-	0.033	Het: 0.84 (0.63 - 1.11)	Ca: 38/169/186
				HM: 0.56 (0.36 - 0.87)	PC: 74/219/202
***LOXL2***	rs1002791	C/+	0.61	Het: 1.05 (0.79 - 1.38)	Ca: 31/155/207
				HM: 1.31 (0.77 - 2.22)	PC: 31/192/268
***LOXL2***	rs17760913	T/-	0.94	Het: 1.04 (0.78 - 1.38)	Ca: 25/148/217
				HM: 1.09 (0.62 - 1.91)	PC: 35/189/266
***LOXL2***	rs17760943	A/-	0.86	Het: 0.96 (0.72 - 1.27)	Ca: 25/139/229
				HM: 0.87 (0.50 - 1.49)	PC: 29/171/293
***LOXL2***	rs2294125	G/+	0.43	Het: 1.01 (0.74 - 1.37)	Ca: 87/196/109
				HM: 1.25 (0.85 - 1.83)	PC: 93/258/144
***LOXL2***	rs3808522	G/-	0.54	Het: 0.85 (0.63 - 1.16)	Ca: 88/176/129
				HM: 0.97 (0.67 - 1.41)	PC: 105/240/151
***LOXL2***	rs3808536	C/+	0.92	Het: 1.01 (0.74 - 1.37)	Ca: 82/188/123
				HM: 1.08 (0.74 - 1.57)	PC: 98/239/157
***LOXL2***	rs6985160	T/-	0.16	Het: 0.77 (0.58 - 1.02)	Ca: 50/165/178
				HM: 1.00 (0.65 - 1.55)	PC: 56/239/200
***LOXL2***	rs6999447	T/-	0.31	Het: 1.25 (0.93 - 1.66)	Ca: 54/184/155
				HM: 1.06 (0.71 - 1.60)	PC: 71/206/217
***LOXL4***	rs11189510	A/+	0.46	Het: 1.21 (0.88 - 1.65)	Ca: 6/100/287
				HM: 1.34 (0.43 - 4.20)	PC: 6/109/381
***LOXL4***	rs11189513	G/-	0.65	Het: 0.91 (0.69 - 1.21)	Ca: 37/162/194
				HM: 0.82 (0.52 - 1.31)	PC: 53/210/229
***LOXL4***	rs11599085	C/+	0.08	Het: 0.74 (0.55 - 0.99)	Ca: 54/160/178
				HM: 0.72 (0.48 - 1.08)	PC: 79/227/186

Adjusted p-value includes gender as a covariate. Because there were 14 SNPs assessed in this step, a Bonferroni correction would lead to significance with p < 0.004. The p-value displayed indicates the significance of the overall model and is adjusted for gender. The odds ratios use the homozygous major allele genotype as the reference genotype and genotype counts are presented for each group.

A secondary analysis of traditional allele association was performed for the SNPs genotyped in the replication cohort. The results indicate that no SNPs were significantly correlated after accounting for multiple testing.

Retrospective power calculations with performed with measured allele frequencies. Our discovery cohort of 138 cases and 411 controls had allele frequencies ranging from 0.06 - 0.49. We had 80% power to detect minimum odds ratios in the range of 1.7 - 2.1 using a 2 degree of freedom genotypic test with alpha = 0.1 (cutoff for inclusion in replication set). Assumptions included genotyping of the causative SNP, a dominant model without additive or multiplicative effects, and a population prevalence of 3%. In the replication cohort of 400 cases and 506 controls, the measured allele frequencies ranged from 0.13 - 0.46. This allowed for 80% power to detect minimum odds ratios in the range of 1.5 - 1.6 with the same parameters. The detection limit increased to 2.6 - 3.6 with a Bonferroni correction and alpha = 0.00357. Note the lowest minor allele frequency measured in the discovery cohort was 0.06, with minimum detectable odds ratio of 2.0. No SNPs carried forward in the replication set had a measured allele frequency of less than 0.13, indicating that rare polymorphisms were not fully assessed in this study.

Additional polymorphisms are added to public databases such as dbSNP with each build. To assess for the extent of coverage, the successfully genotyped SNPs were tested as proxy markers for all SNPs with allele frequency > 0.1 within 100 kb of each lysyl oxidase gene included in the 1000 Genomes pilot data release. We achieved an average r^2 ^of 0.76 over 131 SNPs in *LOX*, 0.68 over 404 SNPs in *LOXL1*, 0.60 over 741 SNPs in *LOXL2*, 0.36 over 224 SNPs in *LOXL3*, and 0.64 over 344 SNPs in *LOXL4*.

## Discussion

Although work in model organisms suggests a role for lysyl oxidases in scoliosis, common variants in the five human lysyl oxidase genes did not show significant association with the adolescent idiopathic phenotype.

These negative results do not provide support for the underlying hypothesis that lysyl oxidases are involved in the development of adolescent idiopathic scoliosis. The mechanisms by which lysyl oxidase enzymes putatively influence scoliosis in experimental models show effect modification, specifically with copper exposure. Prior work demonstrated that some mutations in a zebrafish model did not overtly cause the abnormal phenotype, but instead allowed the expression of the phenotype at previously subclinical levels of copper deprivation [14]. Likewise, the incidence and severity of scoliosis in the genetically predisposed chickens were sensitive to dietary copper [15,16]. Measures of copper intake or homeostasis were not available in this population.

The inherent limitations of this study must be acknowledged. Although we studied a large combined cohort of patients with AIS, this study was not powered to detect small risks. We recognize that in addition to sample size, the minor allele frequency of a particular polymorphism and the linkage disequilibrium between the putative causative variant and the genotyped SNP are the primary driving forces behind the detectable genotype relative risk. In addition, the analysis was performed without predefining a specific model such as additive or multiplicative. We selected a genotypic model which uses two degrees of freedom, resulting in lower power because it allows for the genotypes to have a relationship other than additive or multiplicative. The use of an unscreened control population with an assumed AIS prevalence of 3% also decreased the effective power of this study, and had the potential to lead to misclassification bias.

Only tag SNPs with a minor allele frequency above 0.10 and previously described coding SNPs were included in this analysis. As a result, only variants which are relatively common in the population were evaluated. The post hoc evaluation of coverage indicates that additional common polymorphisms in these genes were not well captured in this study. Since newly reported variants were not all sufficiently tagged, this study does not address their potential to have association with AIS. Additionally, a phenotype with complex inheritance such as adolescent idiopathic scoliosis may be due relatively rare, but more penetrant, variants and these were not examined in this study.

## Conclusions

Common polymorphisms in the lysyl oxidase family of genes were not found to associate with the phenotype of adolescent idiopathic scoliosis. These results suggest future research in a number of different directions. The lysyl oxidase genes could be examined in other idiopathic scoliotic phenotypes. Adolescent idiopathic scoliosis has onset with puberty, but the lysyl oxidases may impact spinal development during earlier windows resulting in congenital scoliosis or juvenile scoliosis. Alternatively, adolescent idiopathic scoliosis may be the appropriate phenotype, but the impact is mediated through individually rare mutations that have a large impact on the overall phenotype in a subset of patients. Further study of rare variants would necessitate sequencing individuals with adolescent idiopathic scoliosis for variants in at least exonic regions, a timely and more costly approach.

## Competing interests

The authors declare that they have no competing interests.

## Authors' contributions

TLM conceived of the study, selected SNPs for genotyping, performed primary analysis, and drafted the manuscript. CAG, MBD, CAW, JAM, TMM and RM collected biospecimens from participants and characterized the phenotypes. LMM participated in study design, data acquisition, and analysis. All authors read and approved the final manuscript.

## Pre-publication history

The pre-publication history for this paper can be accessed here:

http://www.biomedcentral.com/1471-2350/12/92/prepub

## Supplementary Material

Additional file 1 **Genotyped SNPs in the discovery phase with resulting p-values and odds ratios **Excel spreadsheet of all SNPs (N = 112) submitted for genotyping in the discovery phase (138 case and 411 control samples). The indicated p-value resulted from a logistic regression genotypic model controlling for gender. The odds ratios for each SNP are given in reference to the homozygous major allele genotype. Click here for file
